# *Swiss Cheese* Gene Is Important for Intestinal Barrier, Microbiome, and Lipid Metabolism Regulation in *Drosophila* Gut

**DOI:** 10.3390/ijms262211085

**Published:** 2025-11-16

**Authors:** Ekaterina A. Ivanova, Elena V. Ryabova, Artem E. Komissarov, Elizaveta E. Slepneva, Anton A. Stulov, Sergey A. Bulat, Svetlana V. Sarantseva

**Affiliations:** Petersburg Nuclear Physics Institute Named by B.P. Konstantinov of National Research Centre «Kurchatov Institute», 188300 Gatchina, Russia; ivanova_ea@pnpi.nrcki.ru (E.A.I.); ryabova_ev@pnpi.nrcki.ru (E.V.R.); komissarov_ae@pnpi.nrcki.ru (A.E.K.); slepneva_ee@pnpi.nrcki.ru (E.E.S.); ant.stulov@yandex.ru (A.A.S.); bulat_sa@pnpi.nrcki.ru (S.A.B.)

**Keywords:** *swiss cheese*, *PNPLA6*, *Drosophila melanogaster*, septate junctions, intestinal barrier, lipid metabolism, microbiome

## Abstract

Mutations in the human patatin-like lysophospholipase domain containing the 6 gene *PNPLA6* encode an evolutionarily conserved (lyso)phospholipase, leading to the development of a complex hereditary spastic paraplegia 39 (SPG 39) and a number of rare severe syndromes in humans. Diseases disrupt the functioning of the nervous and reproductive systems and the gastrointestinal tract. The study aims to investigate the role of the *Drosophila melanogaster swiss cheese* gene, an ortholog of the human *PNPLA6* gene, in gut function. We showed that the *swiss cheese* gene knockout leads to changes in the morphology of the midgut, disruption of the septate junction structure and the intestinal barrier permeability, and a decrease in the lipid droplet number in enterocytes. As a result of such disturbances, intestinal stem cells (ISCs) proliferation is activated, and the gut microbiome is altered. Ectopic expression of human *PNPLA6* leads to the recovery of the intestinal barrier in the fly gut. The example of *Drosophila* demonstrates the important role of evolutionarily conserved (lyso)phospholipase in intestinal homeostasis.

## 1. Introduction

Recently, there has been a growing number of scientific papers demonstrating the relationship between the development of chronic, inflammatory, metabolic, neurodegenerative, and autoimmune diseases with gastrointestinal tract dysfunction and intestinal microbiota imbalance (dysbiosis) [1,2,3]. Intestinal dysbiosis in particular can lead to systemic inflammation, disrupt the blood–brain barrier, and produce neuroactive compounds, affecting both the central nervous system and peripheral neurons innervating the intestine [4,5]. For example, gastrointestinal disorders caused by the violations in the functioning of the autonomic nervous system are observed in complex forms of hereditary spastic paraplegia [6]. However, at the moment, the causes of these symptoms are not considered in detail when studying the pathogenesis of this disease and developing therapy.

The evolutionarily conserved *PNPLA6* gene encodes phospholipase B, a member of the patatin-like phospholipases family. It has been shown that PNPLA6 is localized in the endoplasmic reticulum (ER) and is responsible for the breakdown of phosphatidylcholine (PC) and lysophosphatidylcholine (LPC) in the cell [7,8]. Mutations in the human *PNPLA6* gene are associated with the development of a complex form of spastic paraplegia 39 and a number of hereditary human syndromes [9,10,11,12]. Human *PNPLA6* is expressed in various tissues and organs [13], and its dysfunction leads to disruption of not only the nervous system [14,15].

Interestingly, the *PNPLA6* gene belongs to a group of host genes that significantly contribute to the formation of the intestinal microbiota. Using genome-wide association studies (GWAS), it was shown that the biosynthesis of mono-, trans-, and polyphosphates, which play an important role in the formation of bacterial cell walls, is associated with the *PNPLA6* locus [16].

Human orthologs of *PNPLA6* show a remarkable architectural similarity in the primary protein structure in a variety of organisms: bacteria, nematodes, yeast, *Drosophila*, and vertebrates [17]. Moreover, in different animals, mutations in *PNPLA6* exhibited similar neurodegenerative phenotypes, indicating an evolutionarily conserved role of the PNPLA6 family in brain function [7,18,19]. In mammals, the function of the *PNPLA6* gene has been widely studied on *Mus musculus*. Expression of *PNPLA6* begins at 7 days for the embryo and is important for its survival [20]. The gene expression has been detected in the testes, kidney epithelium, and liver [20,21,22]. In the central nervous system, the protein is detected in the pyramidal cells of the hippocampus and in the Purkinje cells of the cerebellum [23]. In the peripheral nervous system, the protein has been found in axons and mature Schwann cells [24]. The neuron-specific *PNPLA6* knockout leads to an increase in the PC level, degeneration of the distal regions of the sensory and motor neurons in the spinal cord, and the death of cells in the hippocampus, thalamus, and the Purkinje cells of the cerebellum [18,25]. The *PNPLA6* knockout in nonmyelinating Schwann cells promotes the incomplete wrapping of nerve fibers by this type of glia and induces the degeneration of peripheral axons [24]. The ubiquitous *PNPLA6* knockout is lethal for mice [20,26].

A huge contribution to understanding the function of the *PNPLA6* gene was made by the fruit fly *Drosophila melanogaster* (*D. melanogaster*), which has an orthologue of the human gene—the *swiss cheese* gene (*sws*). The *sws* gene was discovered by D. Kretzschmar during a study of *Drosophila* mutants with a neurodegenerative phenotype [26]. It was further shown that the *sws* function is necessary for the functioning of neurons and some types of glia, in particular subperineural glia, which are part of the *Drosophila* blood–brain barrier [7,27,28,29,30,31]. Like human PNPLA6, sws contains an esterase domain, and its deletion results in increased PC and LPC [7,8]. In the cell, sws is localized in the ER dysfunction of the catalytic domain and causes disturbances in its structure and functions, apparently due to the increase in phospholipids in the cell [7]. In flies with *sws* gene knockout (*sws^1^*), an increase in cytoplasmic calcium was found, which indicates an impairment of its delivery to the ER and the expression of *SarcoEndoplasmic Reticulum Calcium ATPase* (SERCA). The expression of *SERCA* and the transcription factor *XBP^1^* during *sws* dysfunction reduce the ratio of LPC/phosphatidylethanolamine (PE) levels, but not PC/PE, and also reduce neurodegeneration and restore the locomotion activity of flies. Therefore, this may indicate that various pathological processes in the body are caused by increased levels of LPC rather than PC [32].

However, changes in the level of phospholipids in the cell can lead not only to disruption of processes inside the cell, which lead to its death, but also affect the assembly of protein complexes in the membrane [33,34,35].

In this work, we focused on studying the role of the *sws* gene in the enterocytes of *Drosophila* gut, in which, in addition to the nervous system, the *sws* gene is expressed. Knockout of the *sws* gene led to change in the midgut morphology due to the disrupted septate junction structure, active proliferation of ISCs, depletion of neutral lipids, and altered microbiome. In this case, enterocytes do not die by apoptosis and remain functional. These data demonstrate for the first time the important role of the *sws* gene in intestinal homeostasis.

## 2. Results and Discussion

### 2.1. Knockout of the sws Gene Results in Altered Midgut Morphology Due to Disruption of the Septate Junction Structure

**Figure 1:**
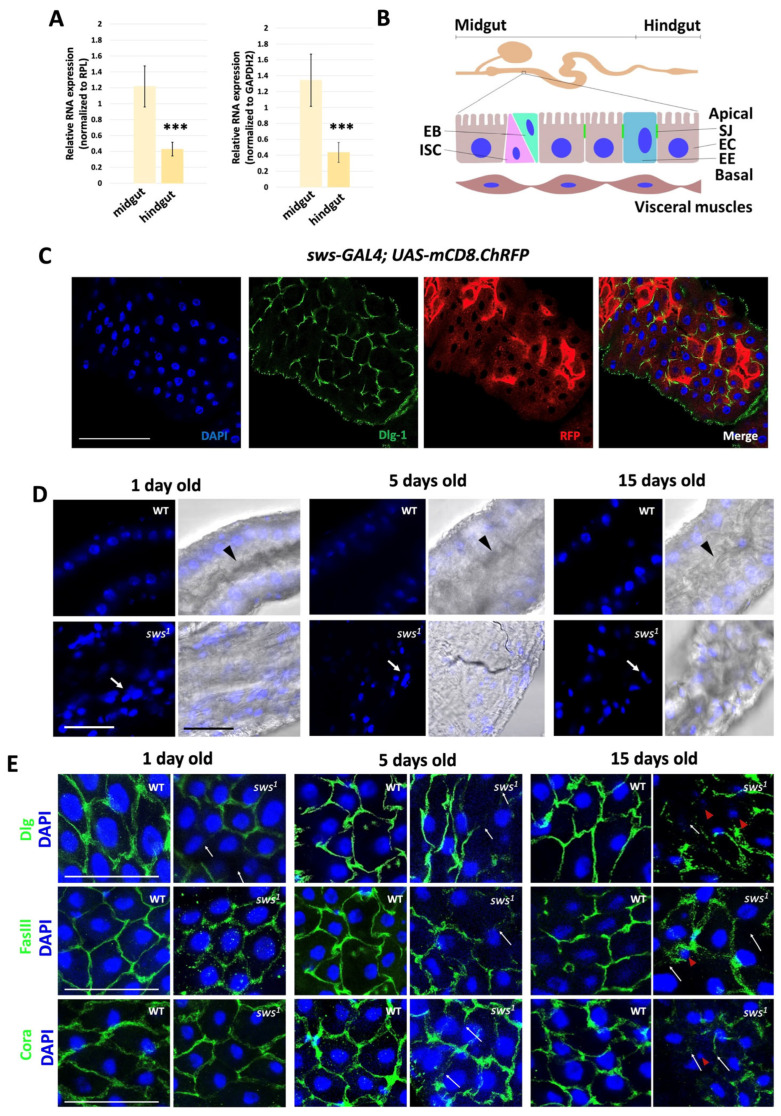
The sws dysfunction in enterocytes violates the cellular organization in midgut of adult flies. (**A**) Relative mRNA *sws* expression in wild type in midgut and hindgut. The mean ± 95% CI, Student’s *t*-test. ***—*p* ≤ 0.001. N = 11. (**B**) Scheme of the *Drosophila* gut. (**C**) Visualization of mCD8.ChRFP in cells with *sws* expression. Green—Dlg-1; red—mCD8.ChRFP; blue—DAPI. Scale bar: 50 µm. (**D**) Cellular organization of the adult fly’s midgut epithelium in wild type (WT) and *sws^1^*. Blue—DAPI and light microscopy. Black arrowheads show the lumen of the intestine. White arrows show violations of the location of the nuclei. Scale bar: 25 µm. (**E**) Visualization of septate junction in *Drosophila* midgut. Blue—DAPI, green—Dlg-1, Cora, FasIII. White arrows show violations of septate junction between enterocytes. Red arrowheads show violations of the location of the nuclei. Scale bar: 25 µm.

In the first stage of the work, we generally confirmed and then studied in detail the expression pattern of *sws* in the midgut, using the RT-PCR (Figure 1A). The intestinal epithelium of *Drosophila* is formed by cell types that differ in origin and function, ISCs, Enteroblasts (EBs), Enterocytes (ECs), Enteroendocrine (EE) cells, and surrounded by a thin layer of muscle cells (Figure 1B). Therefore, it was important to determine which of these cells express *sws*. For this purpose, the activity of the *sws* promoter in the fly intestine was analyzed using the transgenic construct *sws-GAL4;UAS-mCD8.ChRFP*. Visualization of cells with *sws* promoter activity is possible as a result of the specific synthesis of the chimeric transmembrane protein mCD8.ChRFP. Additionally, the boundaries of enterocytes in the intestine were visualized using the septate junction marker disks large 1 and DAPI as nuclear markers of all intestine cells. The red fluorescent protein signal is located within cells that border septate junctions and have large nuclei, indicating *sws* expression in intestinal enterocytes (Figure 1C). Enterocytes of the midgut absorb and transform nutrients such as sugars, amino acids, lipids, and vitamins within the cell. By forming septate junctions between cells, enterocytes maintain the intestine barrier that ensures the transport of substances and protects against the penetration of pathogens into the fly’s hemolymph.

Intestinal morphology was assessed during the period of *sws* expression in the intestine, from day 1 to day 15 of the days old fly [36] using parameters such as the presence of a lumen in the intestine and the location and shape of enterocyte nuclei. Figure 1D shows that in the WT of different ages, the enterocytes nuclei are arranged in a single layer and maintain the same size; the intestinal lumen, where food enters, is clearly defined. In the *sws^1^* mutant, already on a 1-day old fly, the number of enterocytes in the midgut increases and their arrangement is disrupted, and there is a decrease in the visualized intestinal lumen. We observe identical changes when the fly is 5 and 15 days old. Such changes in the midgut morphology may result from the activation of ISC proliferation [37]. At the same time, it has been shown that in the *Drosophila* intestine, ISC proliferation may be a consequence of increased production of reactive oxygen species [38]. Therefore, we analyzed the level of reactive oxygen species (ROS) in the intestine using the 2′,7′-dichlorodihydrofluorescein diacetate (H2DCF-DA) probe in midgut lysates. The level of reactive oxygen species in the midgut of *sws^1^* mutants did not differ from WT ones (Figure S1), so we suggest that oxidative stress is not the cause of ISC proliferation in this case.

On the other hand, normal midgut homeostasis is also based on the relationship between the activation of ISC proliferation and the activation of cell death [39], so in the next stage of the work we analyzed apoptotic cell death in the intestine, since it is known that in the nerve and glial cells of *Drosophila melanogaster*, dysfunction of the *sws* gene leads to apoptosis [27,29]. For this purpose, antibodies to caspase-3, which is a marker of apoptosis, were used. Figure S2 shows that in flies of different ages, both in WT and in the *sws^1^*, caspase-3 is detected equally in small quantities throughout the cell area. It was concluded that enterocytes do not die as a result of sws phospholipase dysfunction.

### 2.2. Disruption of the Septate Junction’s Structure Leads to Permeability of the Intestinal Barrier in the sws^1^ Mutant

Septate junctions are known to provide structural integrity and create a physical barrier in the tubular epithelium of the midgut. Disruption of protein complexes of septate junctions can lead to the activation of ISC proliferation [37,40,41]. Therefore, at the next stage of the work, markers of septate junctions were visualized by the immunohistochemistry using specific antibodies to the proteins Dlg-1, FasciclinIII (FasIII), and Coracle (Cora). The Dlg-1 and Cora proteins are important for regulating epithelial cell shape changes, which is necessary for the formation of the tubular structure characteristic of the midgut. FasIII is localized in large aggregates in the apicolateral region of the midgut and is an adhesion protein [42].

The localization of Dlg-1, FasIII, and Cora proteins occurs in the guts of WT and *sws^1^* mutants (Figure 1E). In the *sws^1^* line, on 1, 5, and 15 days old, the proteins of septate junctions are visualized, as in the WT, but have a heterogeneous structure (shown by white arrows), which indicates a disruption in the structure of intercellular junctions. In our opinion, this results in a disruption in the arrangement of enterocytes in the intestinal epithelium (shown as red cones) of *sws* knockout flies compared to WT flies.

**Figure 2:**
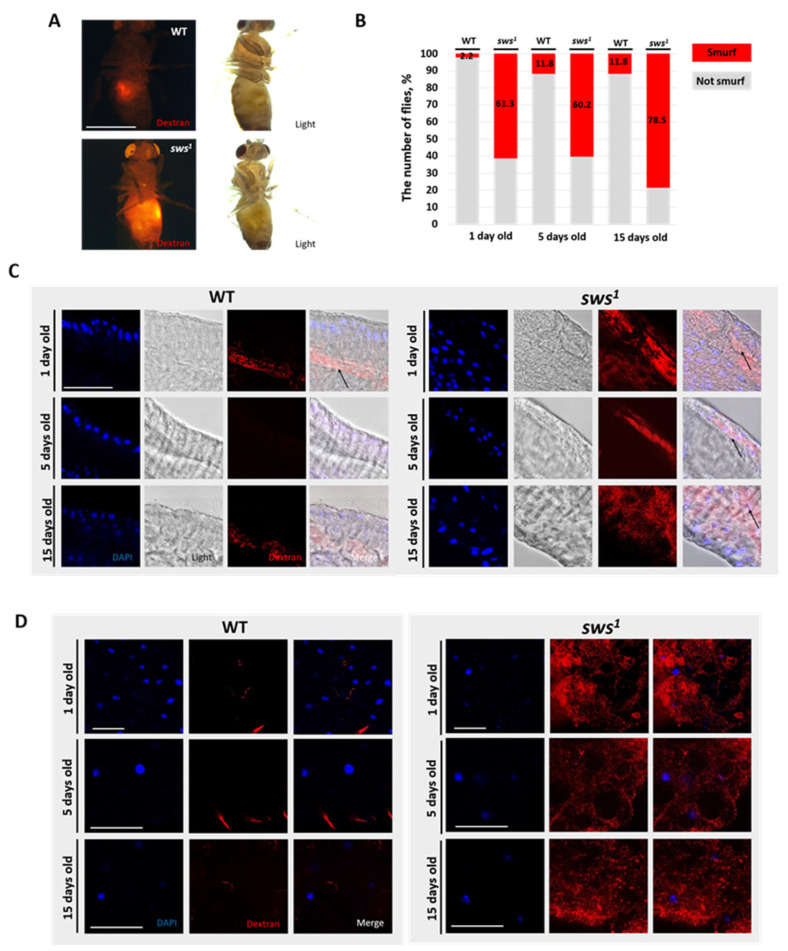
Analysis of the permeability of the intestinal barrier. (**A**) Smurf test. Visualization of dextran 10 kDa, conjugated with Texas red in the fly’s body. Light and fluorescence microscope. Scale bar: 200 µm. (**B**) Stacked bar chart, showing the number of WT and *sws^1^* individuals with a leakage of dextran. (**C**) Dextran visualization in the anterior part of the midgut in WT and *sws^1^*. Blue—DAPI, red—dextran 10 kDa, conjugated with Texas red and light microscopy. Black arrows show the localization of dextran in the gut. Scale bar: 50 µm. (**D**) Analysis of the permeability of dextran in fat body. Blue—DAPI, red—dextran 10 kDa, conjugated with Texas red. Scale bar: 25 µm.

On the other hand, changes in the structure of septate junctions can cause disruption of the intestinal barrier, so its permeability was further analyzed. For this purpose, flies of different ages were fed for 24 h with juice containing 10 kDa dextran conjugated with Texas red (Figure 2A). After 24 h, dextran was visualized inside the flies using fluorescence microscopy. The individuals were divided into two groups: the first group—with dextran localization only within the intestine—and the second group—with dextran localization throughout the body (Figure 2B,C).

Additionally, the distribution of dextran in the gut and fat body was analyzed using laser confocal microscopy. Figure 2C shows that dextran is localized within the intestinal lumen in WT, but in flies with the *sws^1^* mutation, dextran is also detected on the intestinal surface, indicating barrier permeability. If the barrier is permeable, the substance enters the hemolymph, which washes all the insect’s organs. The fat body does not contain septate junctions; therefore, it is permeable to dextran. Fat body analysis showed that dextran was visualized only in the *sws^1^* mutant but not in the WT. As a result of these studies, it can be concluded that dysfunction of the *sws* gene leads to disruption of the intestinal barrier (Figure 2D).

Impaired barrier function in phospholipase sws dysfunction has been demonstrated previously. The sws is necessary for the viability of subperineural glia, the death of which disrupts the integrity of the insect’s blood–brain barrier [28,30]. The *sws* gene is known to control the levels of phospholipids in the cell, which are known to influence membrane protein complexes, namely their activity, stability, and biogenesis [35,43]. Apparently, changes in lipid composition affect the stability of protein complexes at septate junctions. This, in turn, leads to the activation of ISC proliferation in the intestine [44].

### 2.3. Ectopic Expression of the Human PNPLA6 Gene Against the Background of the sws^1^ Mutation (Rescue Experiments)

**Figure 3:**
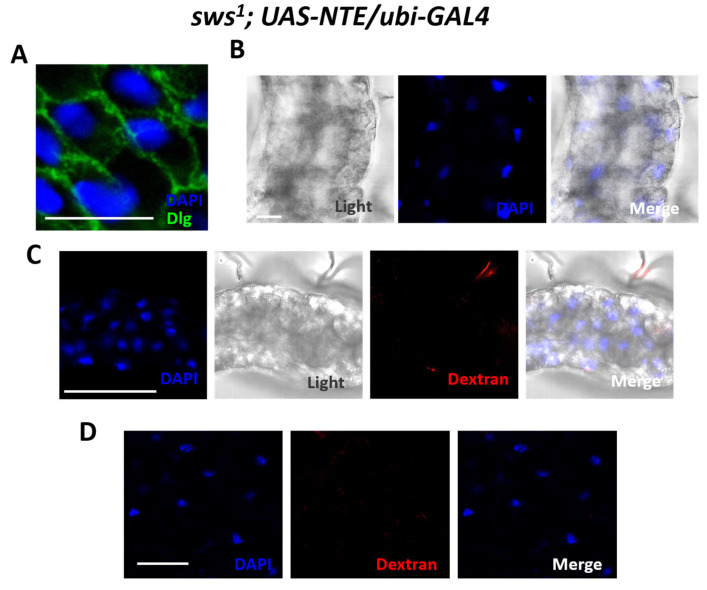
Morphology of midgut with ectopic expression of human *PNPLA6* gene against the background *sws^1^* mutant (*sws^1^;UAS-NTE/ubi-GAL4*). (**A**) Visualization of septate junction in midgut. Blue—DAPI, Green—Dlg-1. Scale bar: 10 µm. (**B**) Cellular organization of the adult fly’s midgut epithelium. Blue—DAPI and light microscopy. Scale bar: 10 µm. (**C**) Dextran visualization in the anterior part of the midgut. Blue—DAPI, red—dextran 10 kDa, conjugated with Texas red and light microscopy. Scale bar: 50 µm. (**D**) Analysis of the permeability of dextran in fat body. Blue—DAPI, red—dextran 10 kDa, conjugated with Texas red. Scale bar: 25 µm.

To prove that the change in the midgut structure in *Drosophila melanogaster* is induced by *sws* knockout, we conducted a rescue experiment. For this purpose, ectopic expression of the human *PNPLA6/NTE* gene was carried out against the background of the *sws^1^*. Next, the morphology of enterocytes, structure of septate junctions, and the functionality of the intestinal barrier were assessed. Compared to the mutant *sws^1^*, the nuclei of 5-day old *sws^1^;UAS-NTE/ubi-GAL4* genotype flies have the same morphology as those of the WT, and the lumen is clearly visible (Figure 3B). Septate junctions are also clearly visualized (Figure 3A). Analysis of the intestinal barrier function showed that dextran is localized only in the lumen and is not visualized in the fat body; that is, the intestinal barrier functions normally (Figure 3C,D). This experiment shows that the function of phospholipase PNPLA6/sws is required for normal intestinal barrier functionality.

### 2.4. Dysfunction of the sws Gene Leads to a Disruption of the Supply of Neutral Lipids in Enterocytes, but Does Not Affect the Absorptive Function

Another key function of the enterocytes of the anterior *Drosophila* midgut is the absorption of nutrients, in particular neutral lipids such as sterol esters, triacylglycerides (TAGs) and diacylglycerides (DAGs), proteins, and glucose. TAG is the most common energy source in insects [45]. Lipid breakdown products (mainly free fatty acids, glycerol, and DAG), along with sterols, are absorbed by enterocytes in the lumen of the midgut through diffusion or specific mechanisms. These lipids are then present in the cell as parts of lipid droplets.

The absorptive function of enterocytes was assessed by measuring the level of glucose and free fatty acids in the feces of WT and *sws^1^*. According to the results of the experiments, the amount of glucose and free fatty acids in the mutant did not change compared to the WT (Figure S3). Therefore, it can be concluded that dysfunction of the *sws* gene does not affect the absorptive function of intestinal enterocytes.

**Figure 4:**
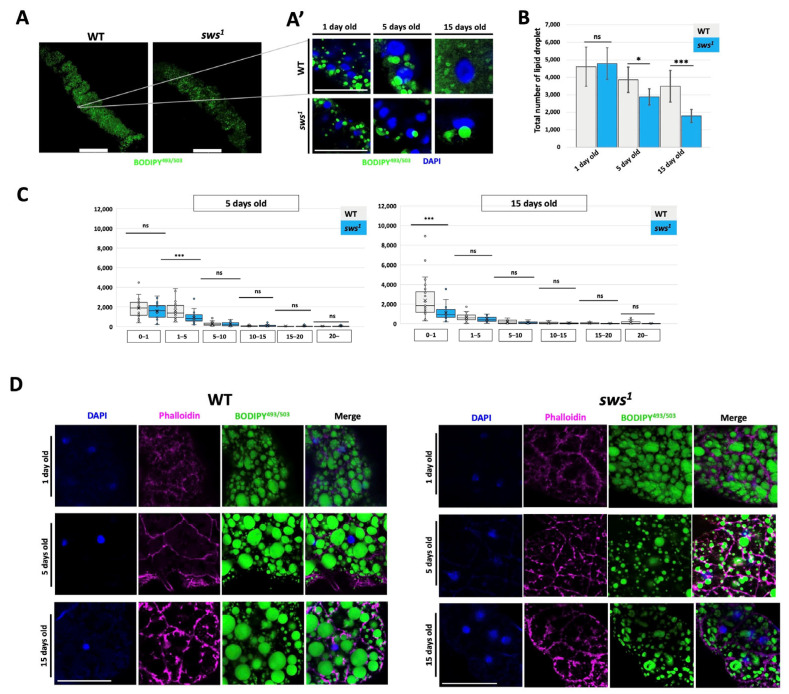
The sws dysfunction reduces the accumulation of lipid droplets in enterocytes of midgut and adipocytes of fat body. (**A**) Visualization of lipid droplets in enterocytes of the anterior part of the midgut in 5-day old flies. Scale bar: 100 µm. (**A’**) Visualization of lipid droplets in enterocytes. Blue—DAPI, green—BODIPY^493/503^. Scale bar: 10 µm. (**B**) The total lipid droplet number in midgut. Mean ± 95% Cl. ns—no significant difference (*p* > 0.05) * *p* < 0.05, *** *p* < 0.001. N = 30. (**C**) Distribution of lipid droplets size (μm^2^) in enterocytes. Mean ± 95% Cl. *** *p* < 0.001, ns—no significant difference (*p* > 0.05). N = 30. (**D**) Visualization of lipid droplets in adipocytes. Blue—DAPI, green—BODIPY^493/503^, magenta—phalloidin labeled by rhodamine. Scale bar: 25 µm.

After absorption from the intestinal lumen, free fatty acids (FFAs) are stored in the form of lipid droplets. The total number and size distribution of lipid droplets in enterocytes were analyzed. For their staining, a specific dye for neutral lipids BODIPY^493/503^ was used. The anterior part of the midgut, where lipid storage occurs, was analyzed in flies of different ages (Figure 4(A,A’)). The number of lipid droplets decreased in flies 5 and 15 days old with the *sws* gene knockout compared to the control (Figure 4B). Analysis of the distribution of the lipid droplets number by size showed a decrease in the number of lipid droplets whose size ranged from 0.1 to 1 μm (Figure 4C).

Since the anterior part of the midgut is immersed in the fat body, into which lipid droplets are transported, their number in the adipocytes of the fat body was further analyzed. In the *sws^1^*, adipocytes are found to have fewer lipid droplets, and they are also smaller in size compared to the WT (Figure 3D). It is known that lipid droplets in enterocytes are formed as a result of the absorption of lipids from the intestinal lumen, as well as a result of the absorption of carbohydrates and the synthesis of lipids from them de novo [46]. From the results obtained in this study, it can be assumed that it is the second process that is disrupted—the synthesis of lipids de novo; in addition, it is known that the product of the *sws* gene is a phospholipase, the enzymatic activity of which determines the intensity of lipid metabolism, and, as a consequence, the accumulation of lipid droplets. Such changes with the accumulation of lipid droplets in the fat body may be associated primarily with a decrease in lipid synthesis in enterocytes.

### 2.5. The Impact of sws Gene Dysfunction on the Gut Microbiome of Drosophila melanogaster

Disturbances in lipid metabolism and proper intestinal functioning can lead to changes in the microbiome. We studied the microbiome of 5-day old flies, since by this age the flies have developed a stable intestinal microbiota [47,48].

**Figure 5:**
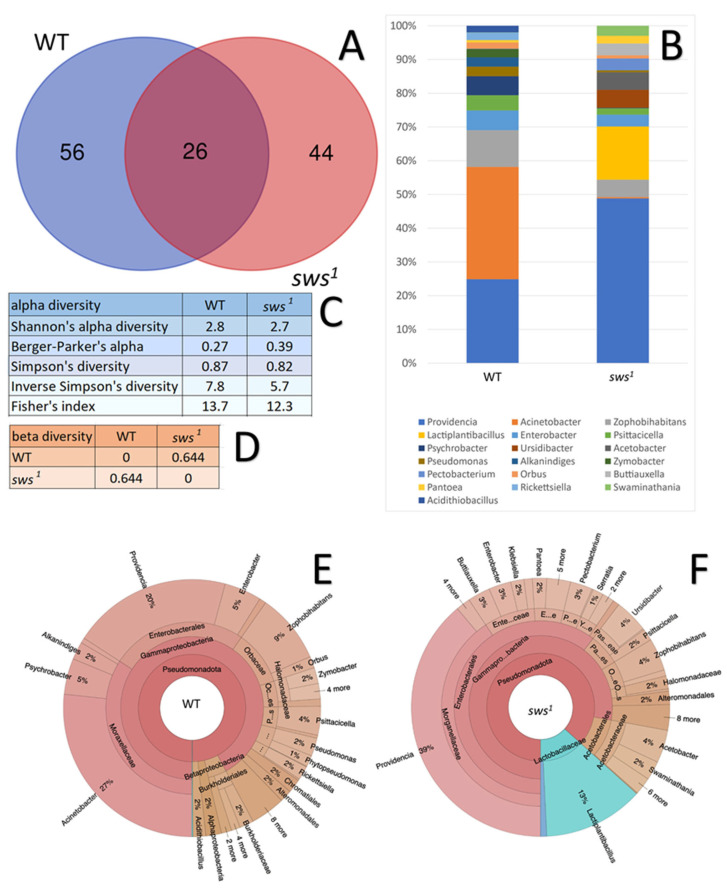
Analysis of 16S RNA amplicon sequencing results. (**A**) Venn diagram illustrating the number of unique and common taxa between WT and *sws^1^* microbiomes. The number inside the intersections represents the number of common genera, and the number outside the intersections shows the specific genera for each group. (**B**) Taxonomic diversity of the gut microbial community in WT and *sws^1^*. (**C**) Comparison of the alpha-diversity indexes between WT and *sws^1^*. (**D**) Beta-diversity of microbiota according to the Bray–Curtis index between WT and *sws^1^*. (**E**,**F**) Krona chart, showing the gut microbial diversity in WT and *sws^1^*.

The data, visualized in a Venn diagram (Figure 5A), show the differences in microbial diversity between the studied groups of 5-day old flies of WT and *sws^1^*. The most significant overlaps are observed among 26 taxa that are found in both genotypes regardless of the *Drosophila melanogaster* line, which corresponds to the basic core of the microbiota. Flies of the *sws^1^* line are characterized by the number of unique bacteria amounting to 44 taxa and a divergence of bacterial diversity from WT by 56 taxa (Table S1).

This study assessed the differences between the microbial diversity in WT and *sws^1^* guts. Figure 5C shows an important characteristic as alpha-diversity This characteristic can be described as a measure of the diversity of a microbial community, which makes it possible to evaluate such parameters as the number of identified species included in the studied communities, the uniformity of their distribution, and in some cases the phylogenetic relationship between them. The analysis of alpha-diversity has included the calculation of several metrics; the most common of them is the Shannon index, which simultaneously reflects not only the total number of taxa present in the sample, but also how evenly the detected microorganisms are distributed among them. As the value of the detected microorganisms is increased, the value of the Shannon index is increased. The Berger–Parker index is characterized by how strongly one taxon dominates the microbial community. Its value decreases as the number of unique taxa in the sample increases. The Simpson index is characterized by the probability that two microorganisms randomly selected from a sample will turn out to be representatives of the one species. If its value is close to zero, then it is considered that the diversity is very high. The Fisher index is a numerical value that makes it possible to assess biodiversity based on the assumption that the number of organisms of different species in the sample is distributed according to a logarithmic model. Its high values allow us to conclude that there are definitely many rare species with a small number of individual microorganisms in the studied community. Also, low values are associated with the dominance of several of the most common species and low species diversity [49].

An analysis of alpha-diversity has shown that WT and *sws^1^* flies are characterized by approximately equal values of most indexes, which indicates a coincidence in the distribution of their microbiota. However, in *sws^1^* flies, the values of most indexes are reduced compared to WT, which implies a relatively simple and less uniform microbial community of microbiota.

In addition to alpha-diversity, beta-diversity is another important characteristic that allows us to assert the presence of significant differences in the composition of the microbiota. This parameter allows us to assess the degree of differences in the composition of species between different samples and habitats. It shows how microbial communities differ from each other, reflecting changes in species composition in space or under the influence of various environmental factors. Similarity and difference indexes such as the Jaccard index, Whittaker beta-diversity, or Bray–Curtis metric are used to quantify beta-diversity [50]. Figure 5D shows the values of beta-diversity between WT and *sws^1^* flies calculated based on the Bray–Curtis metric, since this index is considered the most representative for analyzing the amplicon sequencing results [51]. The elements outside the diagonal reflect numerical differences between pairs of samples: the closer the value is to 0, the more similar the microbial community; the closer to 1, the higher the difference.

The analysis of beta-diversity has shown that the microbial communities of the WT and *sws^1^* guts differ slightly from each other in the diversity of the detected taxa (Figure 5E,F).

The microbiome of WT and *sws^1^* flies, in its composition and structure, generally corresponds to up-to-date data on the diversity of the intestinal microflora of *Drosophila*. Among the most significant and predominant bacteria taxa are the genera *Acinetobacter*, *Providencia*, *Enterobacter*, *Psychrobacter*, and *Lactiplantibacillus* (Figure 5B) [52,53,54,55]. It is worth noting that the microbiome of the control line is characterized by the predominance of such key bacterial genera as *Acinetobacter*, *Zophobihabitans*, *Enterobacter*, and *Psychrobacter*. The representatives of the genus *Acinetobacter* are generally described as a classic component of the microbial diversity of the *Drosophila* gut. The species taxa of the genus *Psychrobacter* are also often identified in the analysis of the microbiota of insects, for example, the housefly *Musca domestica* [56], but amongst them there are some pathogens for humans [57].

The change in the intestinal microbial balance in flies with the sws gene knockout can be is characterized by the appearance of bacteria of the *Lactiplantibacillus* species in their microbiome. The species of this genus are generally described as beneficial symbionts of *Drosophila*, important for maintaining the normal physiology of the host [58,59]. Moreover, in a number of works this genus is mentioned in the context of improving the metabolic processes of *Drosophila* associated with digestion, as well as an activator of specific immune pathways [60,61]. In addition, in this study in the microbiome of *Drosophila* with *sws* gene dysfunction several unique species, such as *Acinetobacter gerneri*, *Acinetobacter bouvetii*, *Aromatoleum toluvorans*, *Paraburkholderia fungorum*, *Limnobacter litoralis*, *Rahnella aceris*, and *Gallaecimonas pentaromativorans*, were discovered. Of them, the *Acinetobacter gerneri* was met in wastewater, activated sludge, and treatment facilities [62]. In addition, it could be introduced into samples by insect vectors as shown in forensic medicine papers [63]. *Acinetobacter bouvetii* is also mentioned in the context of wastewater studies, being considered an environmentally resilient species, identifiable even in contaminated sites. Interestingly, *Acinetobacter bouvetii* can reduce oxidative stress induced by the presence of heavy metals (such as chromium) in plants [62,64]. *Aromatoleum toluvorans* is reported in studies of aquatic microbial communities, as well as in soils and activated sludge [65]. It was also mentioned in the studies as dealing with the microbiomes of invertebrates and humans [66]. The bacteria *Gallaecimonas pentaromativorans* have the ability to decompose high-molecular polycyclic aromatic hydrocarbons and contribute to the purification of water systems contaminated with oil compounds [67]. The species *Paraburkholderia fungorum* shows resistance to environmental pollution and could be involved in soil bioremediation [68]. The species *Rahnella aceris* is well reported for the insect microbiome; in particular, there is evidence that it is able to break down a whole range of substrates, including esters, xylan, and lipids. It is a key component of the microbial communities of the bark beetle gut [69].

It is known that the integrity of the intestinal barrier determines the formation of the fly microbiome, and its dysfunction causes a distinct shift in the taxonomic diversity of the intestine [70]. Interestingly, these changes are not determined by the chronological age of the insects studied [71]. The main factors in the emergence of microbial imbalance with changes in the integrity of the intestinal epithelial barrier include disturbances in the local immune function of the intestinal epithelium, leading to an expansion of the niche available for bacterial growth, as well as significant changes in the composition of the nutrient medium that supports or inhibits the growth of certain bacterial strains [72]. In addition, in *Drosophila*, the main mechanism of defense against microbial pathogens is a systemic humoral response aimed at the synthesis and release of antimicrobial peptides by fat body cells [73]. It has previously been shown that knockdown of sws in subperineural glial cells causes an increased inflammatory response [31].

To date, numerous studies have been devoted to the study of the contribution of various molecular factors to the development of intestinal immunity in insects [74,75,76,77]. Although there is an opinion that the influence of the host’s innate immunity is insignificant when intestinal microbial homeostasis is stable, it is clear that its contribution to the development of intestinal microflora can be significantly increased by inflammation or activation of the immune system by certain representatives of the microbiota [78].

Studies on *Drosophila melanogaster* have shown that two intracellular signaling cascades can be activated in the fat body cells of fruit flies in response to bacterial infections [79]. The Toll signaling pathway in *Drosophila* is activated in the presence of infection caused by Gram-positive bacteria and fungi, while the IMD (immunodeficiency) signaling pathway is activated predominantly by Gram-negative bacteria [80]. Furthermore, it has been shown in *Drosophila* that the IMD pathway-inhibiting proteins Pirk and PGRP-LB are “sensor” proteins and contribute to the increased specificity of the intestinal immune response [81].

Interestingly, some proteins involved in regulating the Toll signaling pathway’s immune response are expressed predominantly in the intestine of arthropods. For example, studies on *Bombyx mori* have shown that BmToll9-2, which is conserved across many insects, is involved in the local intestinal immune response. This is supported by the fact that this protein is predominantly expressed in the intestine, while its expression level in other organs is significantly lower [79]. Also, studies on silkworm larvae have shown that another Toll pathway receptor protein (BmToll9-1) is expressed predominantly in the midgut tissues of the larvae, likely participating in maintaining their microbial homeostasis [82].

Thus, the obtained data show the influence of *sws* gene dysfunction on the formation of a specific bacterial community. It is likely due to the disruption to the epithelial barrier, which facilitates the colonization of the intestine by a specific set of bacteria that are rarely found in WT strains, and this may be led to changes in metabolism and the immune response in these flies.

## 3. Materials and Methods

### 3.1. Drosophila Stocks and Feeding

Flies were maintained on standard semolina media. Crosses were cultured at 25 °C (12 h night/12 h day). Only male flies were analyzed in all experiments. In this work, the binary Gal4/UAS system was employed.

For visualization of the *sws*-specific gut cells, the y[*],w[*],P{w[+mW.hs]=GawB}sws[NP4072]/FM7c line (KYOTO DGGR #104592, hereinafter abbreviated as *sws-GAL4*, kindly donated by Halyna Shcherbata) and the fluorescent reporter of w^*^;P{UAS-mCD8.ChRFP}3 line (BDSC #27392, hereinafter abbreviated as *UAS-mCD8.ChRFP*) were used.

To analyze consequences of *sws* dysfunction, *sws^1^* is a null allele (as described in [27], kindly donated by Doris Kretzchmar) and control line of *Canton S* (St. Petersburg State University fly collection, kindly donated by Elena Golubkova, hereinafter abbreviated as wild type) was used.

To induce human *PNPLA6* gene ectopic expression, *w^1118^; p[PUAST]-hNTE/CyO* line (hereinafter abbreviated as *UAS-NTE,* (kindly donated by Robert Wessells)) was used. For induction of ubiquitous expression, w[*]; P{w[+m*]=Ubi-GAL4.U}2/CyO (BDSC #32551, hereinafter abbreviated as *ubi-GAL4*) was used.

### 3.2. Quantitative Analysis of mRNA Level

For the stage-specific analysis of *sws* gene expression, wild type midguts and hindguts were dissected in cold PBS. Total RNA was extracted from 30 guts/replicate by ExtractRNA (Evrogen, Moscow, Russia). For purification of genomic DNA, 1000 ng of the total RNA was transferred to a separate tube and treated with DNase I (Themofisher, Waltham, MA, USA), according to the standard protocol. The MMLV RT kit (Evrogen, Moscow, Russia) was used for cDNA synthesis according to the manufacturer’s instruction. For the quantitative RT-PCR reaction, 5X qPCRmix-HS SYBR (Evrogen, Moscow, Russia) was used, according to the manufacturer’s instructions. The reaction was carried out in triplicates using the CFX96 thermocycler (Bio-Rad, Hercules, CA, USA). The reaction conditions were as follows: 40 cycles of 95 °C–20 s, 59 °C–15 s, 72 °C–20 s and subsequent melt-curve analysis for verifying the single product presence in each reaction. Primers were common for all known sws transcripts (5′ to 3′): TCACAGCAACGGTGTACATG and TTACGGTGCTAATGGAACGG.

As a reference, RPL and GAPDH2 genes were chosen, and the corresponding primers were the following (5′ to 3′): RPL: ATGCTAAGCTGTCGCACAAATG and GTTCGATCCGTAACCGATGT, GAPDH2: GATGAGGAGGTCGTTTCTAC and ACCAAGAGATCAGCTTCAC. Data analysis of Q-RT-PCR was performed with BioRad CFX Manager 3.1., Hercules, CA, USA Statistical analysis was performed using KyPlot 5.0. software. All samples were tested for normality with the Shapiro–Wilk test. Student’s *t*-test was used in case of normal distribution. Data were presented as histograms (mean ± 95% confidence interval (CI)). Data were presented as boxplots.

### 3.3. Permeability Assay

To analyze the functionality of the gut barrier, flies were kept on agar and fed through thin capillaries containing food for 16 h. An apple juice with dextran 10 kDa, conjugated Texas red (1:50, Invitrogen by ThermoFisher Scientific, Waltham, MA, USA), was used as a food. Then, using a Leica DM2500 fluorescence microscope (Leica, Wetzlar, Germany), the distribution of dextran in the body of flies was analyzed. Next, the number of flies in which dextran was localized in the gut and flies in which dextran was distributed throughout the body was calculated. After that, the fat bodies and guts were isolated in cold PBS with subsequent fixation of 4% paraformaldehyde for 30 min and analyzed on the laser scanning confocal microscope Leica TCS SP5 (Leica, Wetzlar, Germany) using LAS AF software.

### 3.4. Immunohistochemical Staining and Microscopy

A total of 15 guts for each genotype were prepared in PBST solution (0.1% Triton and 1×PBS (BioLine, Saint Petersburg, Russia)) and fixed in 4% paraformaldehyde for 30 min. Next, samples were washed 3 × 5 min in PBS and incubated with primary antibodies diluted in blocking buffer (Visual Protein, BP01-1L, Taipei, Taiwan) at +4 °C overnight. After washing in PBS 3 × 5 min, samples were stained for 2 h at room temperature with a secondary antibody. The guts were washed 3 × 15 min after immunohistochemical staining and placed in a mounting medium with DAPI (Vectashield, Vector Laboratories, Newark, CA, USA). The following primary antibodies were used: mouse 4F3 (anti Dlg-1, 1:100, DSHB, Iowa City, IA, USA), mouse C566.9 (anti Cora, 1:20, DSHB, Iowa City, IA, USA), mouse 7G10 (anti FasIII, 1:20, DSHB, Iowa City, IA, USA), and rabbit Cleaved Caspase 3 (1:400, Cell Signaling Technology, Danvers, MA, USA). The following secondary antibodies were used: goat anti-mouse Alexa 488 (1:400, Abcam, Cambridge, UK) and goat anti-rabbit Alexa 594 IgG1 (1:400, Abcam, Cambridge, UK).

Images of the stained guts were taken using a laser scanning confocal microscope Leica TCS SP5 (Leica, Wetzlar, Germany) using LAS AF software. For image processing, LAS X software was used.

### 3.5. Lipid (FFA) and Glucose Quantification

The absorption function of enterocyte cells was assessed as follows: excrements were collected from wild type and *sws^1^* flies in an Eppendorf tube.

The level of FFAs was assessed using the Free FattyAcids ContentAssay Kit (Solarbio, Beijing, China) according to the manufacturer’s instructions. The absorbance of each well was measured using a Multiskan FC spectrophotometer (Thermo Fisher Scientific, Waltham, MA, USA) at a wavelength of 540 nm.

The level of glucose was assessed using the Glucose (HK) Assay Kit (Sigma-Aldrich, St. Louis, MO, USA) according to the manufacturer’s instructions. The absorbance of each well was measured using a plate reader (EnSpire2300, PerkinElmer, Waltham, MA, USA) at a wavelength of 340 nm.

### 3.6. Lipid Droplet Visualization in Enterocytes and Adipocytes

At least 30 guts and fat bodies for each genotype and age were dissected in cold PBS, fixed in 4% paraformaldehyde for 15 min, and washed in PBS 3 × 10 min. For visualization of lipid droplets, BODIPY^493/503^ (Invitrogen, Thermo Fisher Scientific, Waltham, MA, USA) diluted in dimethylsulfoxide (Vekton, St. Petersburg, Russia) was added in the concentration of 3 mM for 30 min, followed by washing in PBS 3 × 10 min. The fat bodies were additionally stained with phalloidin labeled by rhodamine (1:200, Abcam, Cambridge, UK) for labeling of adipocyte boundaries. Then, the guts and fat bodies were washed in PBS 3 × 10 min and placed in the mounting medium with DAPI (Vectashield, Vector Laboratories, Newark, CA, USA) for the same-day imaging. Series of images (distance between images 3 μm) were obtained with the laser scanning confocal microscope Leica TCS SP5 (Leica, Wetzlar, Germany) using LAS AF software. For image processing, LAS X software was used. For analysis of lipid droplet number, we used the ImageJ software 1.52u (“Analyse Particles” function).

### 3.7. Oxidative Particle Measurement

The ROS level was measured using H2DCF-DA (Invitrogen, Thermo Fisher Scientific, Waltham, MA, USA), as described in [83]. Thirty midguts for each biological replicate, among three total replicates, were homogenized in PBS. The homogenate was centrifuged (for 10 min at 10,000× *g* rpm, +4 °C). Then, 5 µL of clear supernatant was mixed with 60 µL of 5 µM H2DCF-DA and incubated for 60 min at +37 °C. The fluorescence emission of DCF resulting from H2DCF-DA oxidation and hydrolysis was scanned at 485 nm excitation and 530 nm emission with a plate reader (EnSpire2300, PerkinElmer, Waltham, MA, USA). The values obtained for ROS levels were normalized to the protein concentration measured by the standard protocol of the Bradford assay and then were averaged for each of the three biological replicates.

### 3.8. 16S rRNA Sequencing and Analysis

Total DNA was isolated from guts with ZR Genomic DNA-Tissue MiniPrep kit (Zymo Research, Irvine, CA, USA) and was amplified with 16S rRNA bacterial gene v3-v4 region-specific degenerate primers (5′ to 3′) Merk-341F (CCTAYGGGDBGCWSCAG) and Merk-805R (GACTACNVGGGTMTCTAATCC) (Evrogen, Moscow, Russia) for 31 cycles using FastStart polymerase (Roche, Indianapolis, IN, USA) at varying 51–55 °C annealing temperatures. The MinION device equipped with Flow Cell R9.4 (Oxford Nanopore, Oxford, UK) was used for nanopore sequencing. The sequencing run was operated by MinKNOW software, version 25.03.7 (Oxford Nanopore, Oxford, UK). The amplicons were about 485 bp in size. To construct libraries, the corresponding kits were implemented—to repair amplicon ends (NEBNext FFPE DNA repair buffer et repair mix), ligate barcodes (Native Barcoding Expansion 1-12 EXP-NBD104), and then sequencing adapters (Adapter Mix II AMII). All these and further steps (like loading the libraries in a flow cell, etc.) followed instructions provided by Oxford Nanopore Technologies (Oxford, UK). The sequencing was run for 72 h.

The obtained Fast5 files were basecalled at super-accurate and trim-barcode options. Resulting FastQ files (reads) were checked for remaining barcodes, and the primers used for the primary amplification were cut out using Cutadapt (version 5.2) [84]. Low-quality reads were removed using Fastcat (version v0.10.2) [85] with a minimum quality score of 10, minimum read length of 350, and maximum read length of 500 nucleotides. Taxonomic classification was performed with Kraken2 [86] using default parameters and the 16S_ribosomal_RNA database [87] as reference. Species- and genus-level abundances were re-estimated with Bracken [88] using a threshold of three reads per sample; taxa represented by fewer reads were excluded as noise. Taxa detected in negative controls (no-template samples) were classified as contaminants and removed from experimental datasets. Alpha- and beta-diversity metrics were calculated with KrakenTools (version v1.2.1) [89]. Krona charts were generated with Krona [90].

### 3.9. Statistical Analysis

Statistical analysis was performed using KyPlot 5.0. software. All samples were tested for normality with the Shapiro–Wilk test. Student’s *t*-test was used in case of normal distribution. Data were presented as histograms (mean ± 95% CI). For other distribution types, Mann–Whitney nonparametric test was applied. Data were presented as boxplots.

## 4. Conclusions

We showed that the sws protein is required for maintaining lipid homeostasis in the midgut and intestinal barrier function. Disruption of the epithelial barrier contributed to the colonization of the intestine with a particular set of bacteria that are rarely found in WT. Such changes in flies may be accompanied by metabolic and immune response disturbances and increased degenerative processes, which identify interesting future research directions in light of the gut–brain axis. Given the high functional homology 0 *Drosophila melanogaster sws* and human *PNPLA6*, conducting such studies, in our opinion, can contribute to a more detailed understanding of the disease’s pathogenesis caused by mutations in the human *PNPLA6* gene and the development of therapeutic approaches to their treatment.

## Data Availability

The original contributions presented in this study are included in the article/Supplementary Material. Further inquiries can be directed to the corresponding author.

## Supplementary Materials

The following supporting information can be downloaded at https://www.mdpi.com/article/10.3390/ijms262211085/s1.
