# Supplementary material for: Swiss Cheese Gene Is Important for Intestinal Barrier, Microbiome, and Lipid Metabolism Regulation in Drosophila Gut
Source: Int J Mol Sci. 2025 Nov 16;26(22):11085. doi: 10.3390/ijms262211085 (PMC12652016; doi:10.3390/ijms262211085)
Supplement: Supplementary file 1 [file ijms-26-11085-s001.zip › Supplementary materials.pdf]

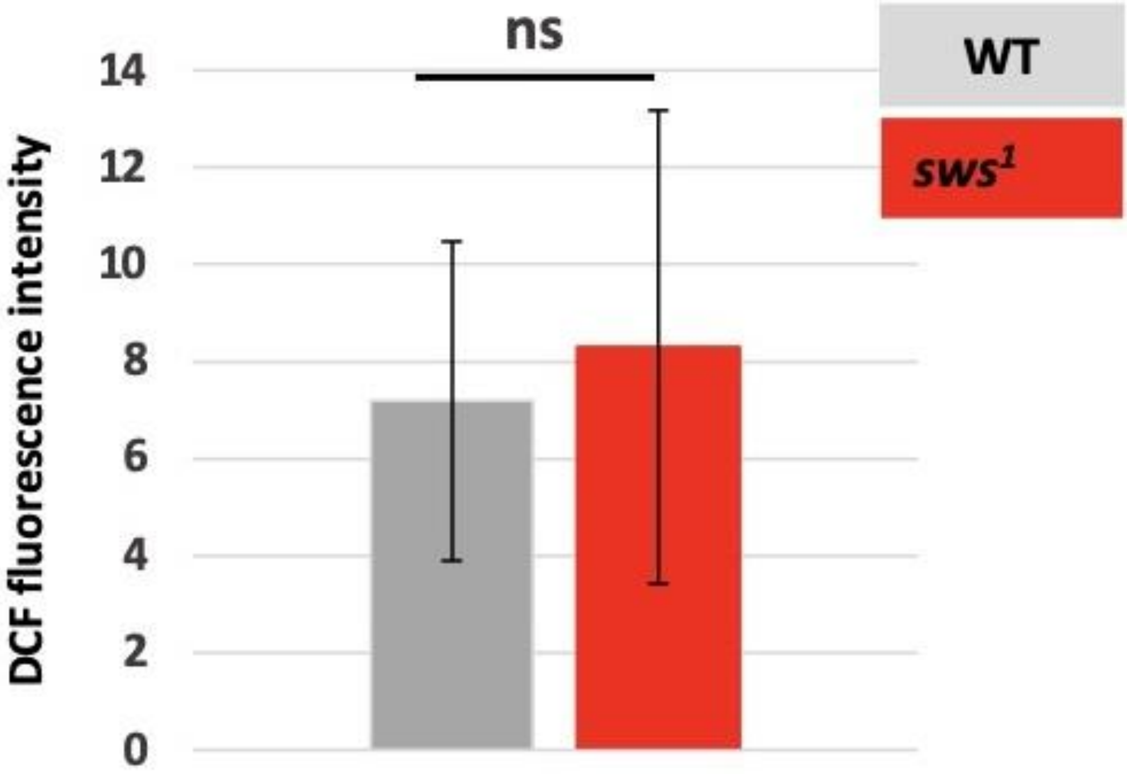

Figure S1: The level of ROS in WT and *sws*[1] midgut

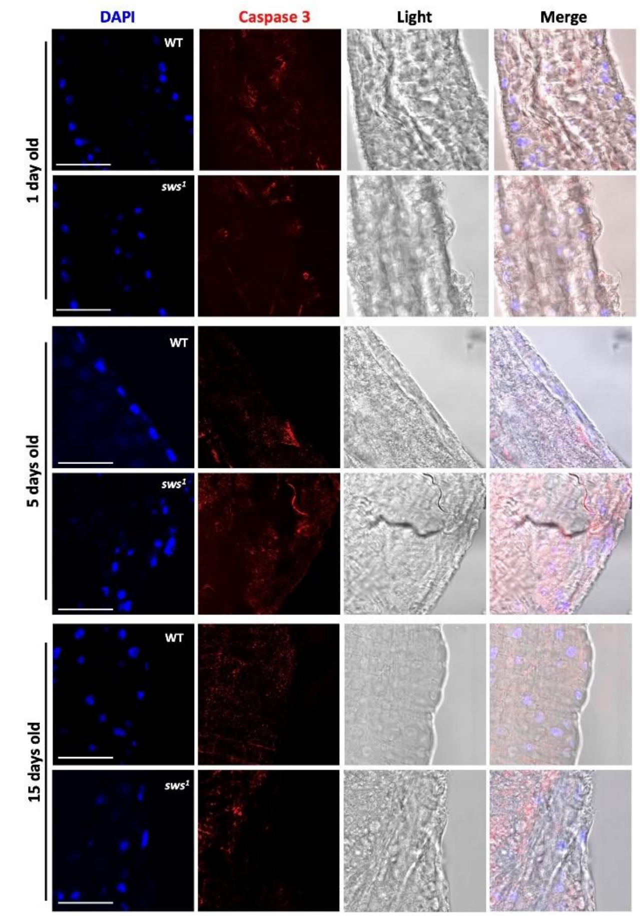

Figure S2: Analysis of apoptosis of midgut in flies of different ages. Scale bar: 25 μm

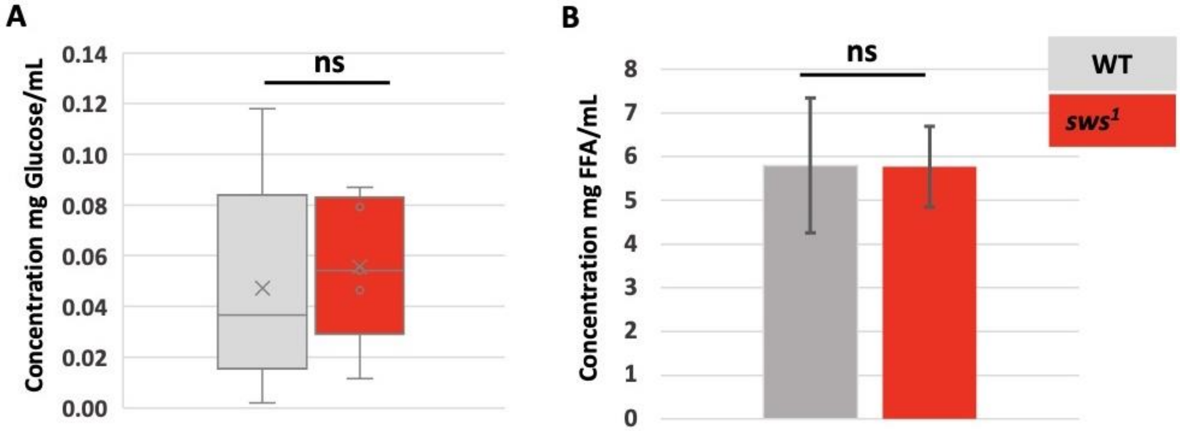

Figure S3: Concentration of glucose and FFA in WT and *sws¹* excrements

Table S1: Generic representation of the metagenome in WT and *sws¹* gut
